# Metropolitan inequalities in health system resources and COVID-19 adjusted life expectancy among older adults in Mexico

**DOI:** 10.3389/fpubh.2026.1807629

**Published:** 2026-05-26

**Authors:** Héctor García-Hernández, Guillermo Salinas-Escudero, Marcela Agudelo-Botero, Hortensia Reyes-Morales

**Affiliations:** 1 Master’s and Doctorate Programs in Medical, Dental, and Health Sciences, National Autonomous University of Mexico, Mexico City, Mexico; 2 Health Research Division, National Institute of Geriatrics, Mexico City, Mexico; 3 Center for Economic and Social Studies in Health, Hospital Infantil de México Federico Gómez, Mexico City, Mexico; 4 Policy, Population, and Health Research Center, School of Medicine, National Autonomous University of Mexico, Mexico City, Mexico; 5 Center for Health Systems Research, National Institute of Public Health, Cuernavaca-Morelos, Mexico

**Keywords:** COVID-19, health resources, healthy-adjusted life expectancy, inequity, life expectancy

## Abstract

**Introduction:**

The mortality and morbidity consequences of the COVID-19 outbreak were concentrated among older adults. The health system response was critical to mitigating its negative effects, particularly through the redistribution of healthcare resources across territories. The objective of this study was to analyze inequities in the distribution of health resources in Mexico in relation to life expectancy (LE) and COVID-19 adjusted life expectancy (CALE) in the 60–64 age group at metropolitan level.

**Methods:**

We conducted an ecological study covering the period from 2020 to 2023. LE was estimated using abridged life tables, and CALE was calculated using the Sullivan method. Correlation analyses were performed to assess the relationships between these indicators and healthcare resource variables. Inequities were quantified using the concentration index, while the dissimilarity index was used to estimate the proportion of resources that would need to be redistributed across metropolitan areas to achieve an equal distribution.

**Results:**

Our findings reveal an unequal distribution of healthcare resources during the pandemic. Metropolitan areas with greater resource availability achieved more favorable health outcomes. This pattern was particularly evident for resources related with specialized services, technological equipment, and health personnel in training. These are precisely the types of resources that should be distributed across metropolitan areas to advance toward a more equitable healthcare system.

**Discussion:**

Resource reallocation becomes a central component of health systems adaptation to public health emergencies. Our results highlight the need for more adequate territorial redistribution of healthcare resources to improve preparedness for future epidemiological emergencies.

## Introduction

The steady improvement in life expectancy (LE) throughout the past century was abruptly reversed by the excess mortality caused by the COVID-19 pandemic beginning in 2020, particularly in low- and middle-income countries and among older adults (1, 2). For example, LE declined by 2.6 years in India (1), and by 1.31 years in Brazil (3). In Mexico, one of the countries with the highest COVID-19 mortality, LE declined by 3.6 years for men and 2.5 years for women in 2020 (4).

However, the consequences of COVID-19 extend beyond mortality, and its morbidity effects remain comparatively understudied (5). Assessing morbidity is essential for understanding the quality of remaining years of life (6), as evidence indicates that it was adversely affected by the COVID-19 pandemic in the short and long term, with older adults identified as the primary contributors to the resulting disability burden (7). Therefore, it’s relevant to analyze the full impact of this condition, which can be captured by health-adjusted life expectancy (HALE) that integrates mortality and non-fatal health outcomes into a single indicator. HALE is typically estimated using disability, although it can be adapted to alternative health metrics, like COVID-19 morbidity, for a more comprehensive assessment of the pandemic’s impact on population health (5).

Moreover, the COVID-19 pandemic exposed and intensified pre-existing inequities, with socially disadvantaged populations experiencing the greatest losses in LE. During the sanitary crisis, income-related disparities were observed, as higher-income groups showed a faster recovery in this indicator (8). Racial inequities were also evident, with larger declines of LE among Hispanic and Black populations compared with White populations in United States (US) (9). Additionally, health disparities were present at the territorial level, where structural disadvantages tend to concentrate spatially and reflect segregation processes driven by ethnic discrimination, unequal market opportunities, and disparities in resource availability (10). For instance, Brazilian northern states experienced the largest declines in LE compared with southern states, where poverty levels are lower (3). A similar pattern was observed in Mexico, where the central and southern regions showed the greatest losses in LE (4).

Urban areas were particularly affected by the pandemic because higher population density and closer interpersonal contact increased the risk of infection compared with rural ones. This risk was especially pronounced in poorer households, where multigenerational living arrangements often led to overcrowding. Further, marginalized populations were the most affected by the loss of millions of jobs resulting from disruptions across multiple industries. These job losses led to deteriorating living conditions, such as limited access to internet connectivity, which further restricted educational and employment opportunities (11, 12). These findings support the recommendation that health disparities should be analyzed at the smallest possible territorial level in order to fully capture existing inequities (13).

The government response to this public health emergency was crucial in mitigating its impact (4). Although responses varied across countries, the performance of health systems constituted a central determinant in preventing mortality and restoring functionality among patients affected by COVID-19 (14). One of the key components of health system performance is the availability of health resources, which its increases are linked to improvements in LE and HALE (15). However, in the context of COVID-19, research examining the relationship between health system resources and LE or COVID-19 adjusted life expectancy (CALE) remains scarce. Only a limited number of studies have addressed this issue, a study conducted in Brazil found that the availability of physicians and intensive care units influenced COVID-19 mortality (16). This topic is particularly relevant, as evidence indicates that a more equitable allocation of health resources can help reduce disparities in health outcomes (17).

Consequently, the objective of this study is to analyze inequities in the distribution of health resources in Mexico in relation to life expectancy and COVID-19 adjusted life expectancy in the 60–64 age group at metropolitan level. By estimating LE and CALE, this study assesses the quantity and quality of remaining years of life affected by COVID-19. While examining their relationship with the distribution of health resources provides critical insight into how territorial variations in healthcare capacity during the outbreak may have contributed to inequities in health outcomes.

## Methodology

We conducted an ecological study covering the period from 2020 to 2023, corresponding to the onset and conclusion of the COVID-19 outbreak in Mexico (18). Four databases were used. Mortality data from the National Institute of Statistics and Geography (*Instituto Nacional de Estadística y Geografía*, INEGI), mid-year population estimates from the National Population Council (*Consejo Nacional de Población*, CONAPO), COVID-19 case data, and information on health resources within the public health system. The last databases were obtained from the General Directorate of Epidemiology (*Dirección General de Epidemiología, DGE*) of the Ministry of Health.

The COVID-19 database includes sociodemographic and morbidity information on confirmed, suspected, and negative COVID-19 cases attended within the public health system since the beginning of the outbreak. It also contains data on admission date, reported symptoms, and laboratory test results. While the health resources database provides information on healthcare personnel, infrastructure, technology, and other general resources available in the public health sector. All databases are publicly available through its corresponding official government websites.

Inequities were analyzed at the metropolitan level. Internationally, a metropolis is defined as an urban territory characterized by high levels of connectivity and economic integration. The criteria commonly used to define metropolitan areas include population density, spatial proximity between urban centers, economic convergence, and commuting flows. In Mexico, 92 metropolitan areas have been officially defined, which comprise 421 municipalities. These metropolitan areas represent the country’s most highly urbanized territories, concentrate a large share of economic activity, and exhibit strong functional integration (11). This level of analysis is appropriate because metropolis were the primary settings in which COVID-19 cases were treated, given that health resources are largely concentrated in these urban areas.

### Variables analyzed

From the COVID-19 case database, we included only confirmed COVID-19 cases. Confirmation was defined according to the official national criteria and included the following scenarios: (1) laboratory confirmation through a positive PCR or antigen test; (2) clinical confirmation, applied when the individual reported close contact with a confirmed COVID-19 case; and (3) confirmation by an expert committee, which was applied to deaths under circumstances in which no PCR or antigen sample was collected, or when a sample yielded an invalid result.

Regarding the public health resources database, we included: first-level and specialty medical offices, hospital beds, intensive care units (ICUs), and emergency departments, all expressed per 100,000 inhabitants; general and family medicine physicians, specialist physicians, physicians in training, and nurses per 1,000 inhabitants; and diagnostic imaging equipment, including computed tomography (CT) scanners and magnetic resonance imaging (MRI) units, expressed per 1,000,000 inhabitants.

### Analysis

Due to the substantial impact of COVID-19 on mortality and disability among older adults, LE was estimated exclusively for the 60–64 age group using abridged life tables with five-year age intervals. Standard life table procedures were applied, as described elsewhere (19). Briefly, this approach requires age-specific mortality rates, calculated using observed deaths and mid-year population estimates. These rates are transformed into age-specific probabilities of death, which are applied to a hypothetical cohort whose members are assumed to experience the observed mortality conditions until all individuals die.

COVID-19 adjusted life expectancy was estimated by adapting the health-adjusted life expectancy to COVID-19 morbidity. As HALE can be constructed using different health outcomes, in this study COVID-19 was used as the disabling condition. Two general approaches are available for this estimation, incidence-based and prevalence-based methods. We employed a prevalence-based approach and estimated CALE using the Sullivan method, which incorporates the prevalence of the health condition into the life table to estimate the expected number of years lived free of COVID-19 (20). To further characterize the morbidity burden, we estimated COVID-19 disability-adjusted life expectancy (DALE) by subtracting CALE from LE, yielding the expected number of years lived with COVID-19-related disability.

Then, we conducted correlation analyses to assess the relations between LE, CALE, and DALE with the health resource variables. Statistical significance was evaluated using a threshold of *p <* 0.05. Subsequently, to examine inequities in the distribution of health resources across metropolitan areas according to the health indicators, we estimated concentration indices for those health resource variables that showed statistically significant relations with the health outcomes.

The concentration index (CI) is a relative measure of inequality that incorporates population size weighting for each analytical unit, producing a single value that reflects the degree and direction of inequality across the entire population. Positive values indicate a concentration of resources among areas with higher health outcomes, whereas negative values indicate concentration among areas with poorer health outcomes. CI values closer to zero indicate equity in distribution. The calculation followed the methodological guidelines proposed by the Pan American Health Organization (PAHO) (21).

Finally, the dissimilarity index (DI) was estimated for health resource variables that were statistically significantly related with LE, CALE, and DALE. This index quantifies the proportion of resources that would need to be redistributed across metropolitan areas to achieve an equal distribution considering its population size and representing a theoretically equitable scenario. DI values range from 0 to 1, where values closer to 1 indicate greater inequality between metropolitan areas, and vice versa. When expressed as a percentage, the DI represents the proportion of total resources that would need to be reallocated for all metropolitan areas to attain the same level of the resource (22).

Analyses were performed using Stata v.17.

This research is part of the project approved under registration number DI-PI-012/2025 by the Research, Ethics, and Biosafety Committees of the *Instituto Nacional de Geriatría*.

## Results

**Figure 1:**
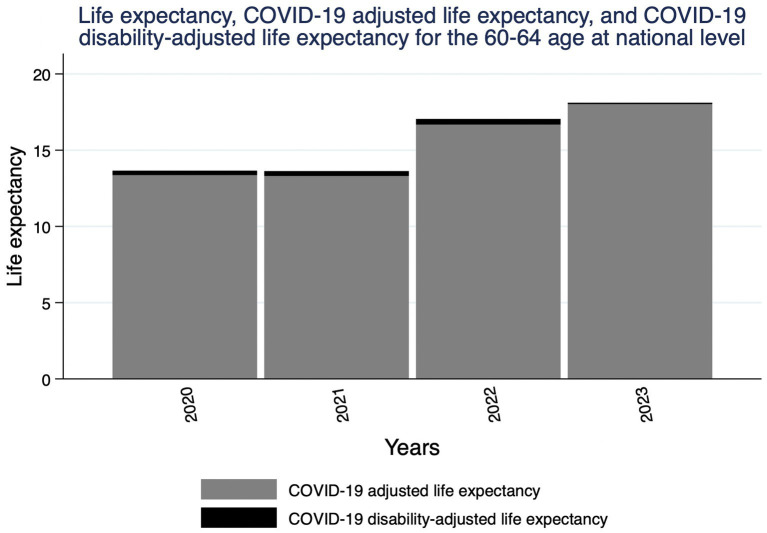
National level life expectancy (LE), COVID-19 adjusted life expectancy (CALE), and COVID-19 disability-adjusted life expectancy (DALE) at ages 60–64 from 2020 to 2023.

Life expectancy, COVID-19 adjusted life expectancy, and COVID-19 disability-adjusted life expectancy for the 60–64 age at national level are presented in Figure 1. In 2020, the impact of COVID-19 mortality is evident in the LE of 13.65 years, followed by a rapid recovery over the study period, reaching 18.10 years in 2023. A similar pattern is observed for both CALE and DALE.

**Figure 2:**
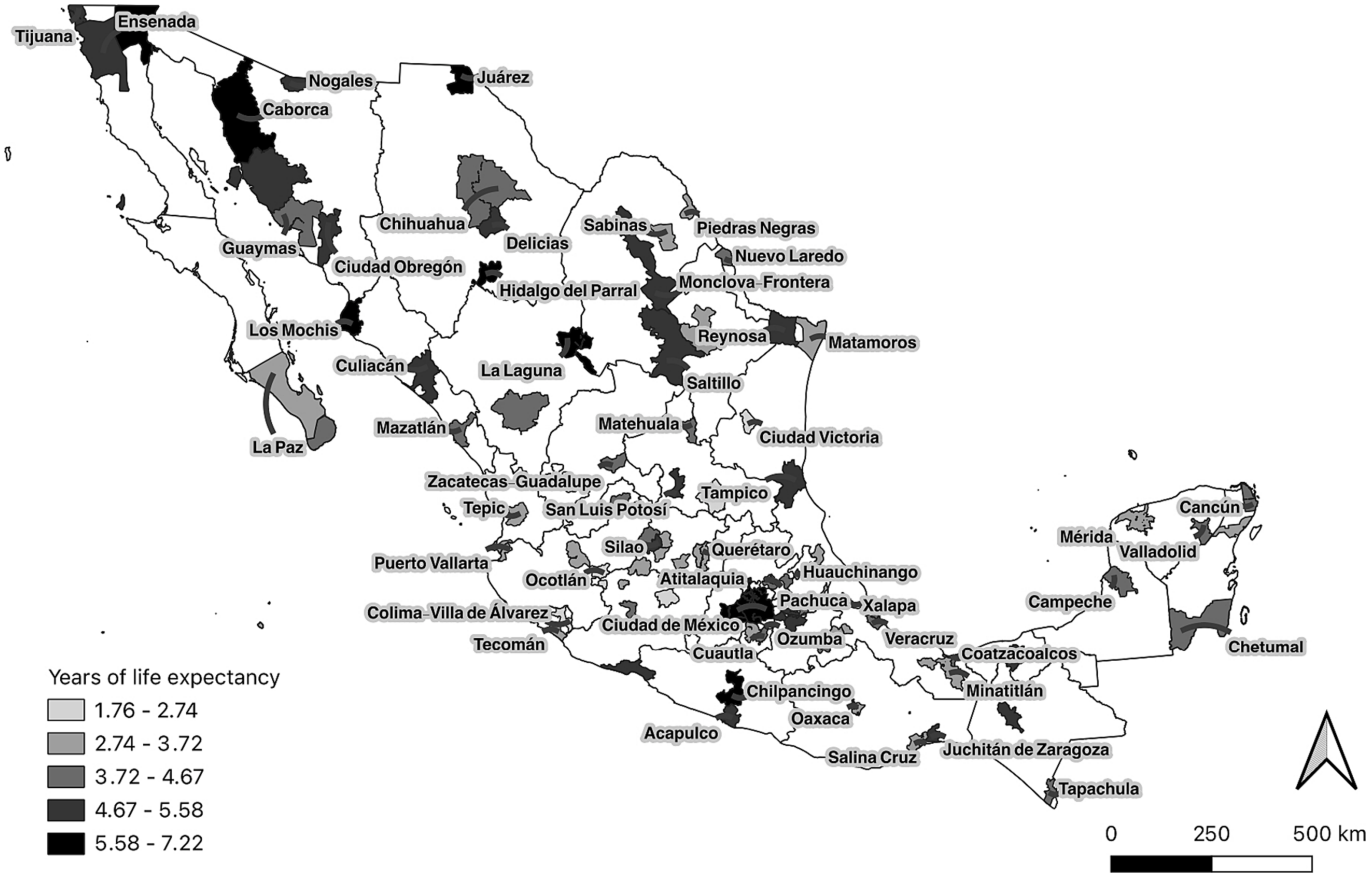
Comparative gains in years of life expectancy from 2020 to 2023 at metropolitan level.

Figure 2 illustrates the comparative gains in years of LE from 2020 to 2023 at metropolitan level. Although substantial heterogeneity across metropolitan areas is evident, the overall recovery trend is consistently reproduced, with a more pronounced improvement in central and northern regions. These patterns reflect the profound impact of the pandemic during its initial phase, followed by a progressive attenuation of its effects by 2023. Estimates of LE, CALE, and DALE at this level are provided in Supplementary material 1.

Table 1 presents the statistically significant correlations (*p* < 0.05) between health system resources and LE, CALE, and DALE across the study period. Blank cells indicate non-significant correlations. Although the magnitude of the correlation coefficients is modest, all reported relationships are statistically significant. The strongest and most consistent pattern is observed in 2021, when all health resource variables were significantly related with at least one health outcome.

**Table 1:** Statistically significant correlations between health resources and life expectancy, COVID-19 adjusted life expectancy, and COVID-19 disability-adjusted life expectancy.

Variable	2020			2021			2022			2023		
	LE	CALE	DALE	LE	CALE	DALE	LE	CALE	DALE	LE	CALE	DALE
Medical offices at first level				−0.24	−0.27							
Medical offices of specialities			0.35			0.48			0.41			0.21
Hospital beds			0.35	0.24	0.21	0.37			0.34			
General and family medicine physicians						0.23						
Specialists physicians	0.22		0.32	0.38	0.34	0.46			0.46			0.35
Physicians in training	0.24	0.21	0.34	0.45	0.41	0.45	0.34	0.25	0.45	0.40	0.39	0.36
Nurses			0.31	0.27	0.23	0.39			0.34			
Computed tomography				0.21		0.32	0.24		0.36			0.28
Magnetic resonance imaging				0.21	0.21							
Intensive care unit						0.24						
Emergency department					−0.21		−0.30	−0.32		−0.29	−0.29	

Statistical significance was evaluated using a threshold of *p* < 0.05.

Physicians in training emerged as the only resource positively related with all health outcomes across all years. In contrast, resources related to the first level of care, namely primary care medical offices and general and family medicine physicians, were generally no related. Notably, negative correlations with lower LE and CALE were observed for emergency departments in 2021, 2022, and 2023.

Table 2 presents the CI and DI for health resource variables that showed statistically significant correlations with health outcomes. Most CI exhibit positive values, indicating that LE, CALE, and DALE are concentrated in more advantaged metropolitan areas, namely those with a higher availability of health resources. In contrast, the negative CI values, apart from those observed for ICUs and emergency departments, such as those for hospital beds and nurses in relation to DALE, are close to zero, indicating little to no health resource related inequity in their distribution regarding health outcomes. The greatest inequities were observed for physicians in training, specialist physicians, MRI units, and emergency departments. Notably, for emergency departments, the negative relationships with health outcomes suggests that these services are concentrated in metropolitan areas with poorer LE and CALE, likely reflecting a higher burden of severe cases rather than deficiencies in service provision.

**Table 2:** Concentration and dissimilarity index for selected health resources variables in 2020, 2021, 2022, and 2023.

Variable	2020				2021				2022				2023			
	Concentration index			DI (%)	Concentration index			DI (%)	Concentration index			DI (%)	Concentration index			DI (%)
	LE	HALE	DALE		LE	HALE	DALE		LE	HALE	DALE		LE	HALE	DALE	
Medical offices at first level					−0.33	−0.33		9.22								
Medical offices of specialities			0.01	7.21			−0.02	7.32			−0.09	7.46			−0.03	7.53
Hospital beds			−0.04	8.21	0.03	0.03	−0.08	8.76			−0.15	8.78				
General and family medicine physicians							−0.34	10.99								
Specialists physicians	0.22		0.14	8.30	0.20	0.20	0.10	8.87			0.02	8.84			0.08	9.19
Physicians in training	0.37	0.38	0.31	12.93	0.33	0.33	0.24	14.22	0.34	0.34	0.15	14.13	0.33	0.32	0.16	13.56
Nurses			−0.01	8.01	0.08	0.09	−0.01	7.83			−0.07	7.97				
Computed tomography					0.12		0.04	17.50	0.13		−0.02	17.65			−0.02	18.08
Magnetic resonance imaging					0.38	0.37		25.58								
Intensive care unit							−0.34	15.16								
Emergency department						−0.46		18.98	−0.46	−0.46		18.92	−0.36	−0.36		16.94

DI values for 2021, 2022, and 2023 indicate that highly specialized services, such as ICUs and emergency departments, as well as medical technology (MRI and CT scanners) were the most inequitably distributed health resources. Among these, MRI showed the highest level of inequity, with a DI of 25.58%. In addition, health personnel also exhibited an inequitable distribution throughout the 2020–2023 period.

## Discussion

To the best of our knowledge, this is the first study in Mexico to examine inequities in the distribution of healthcare resources across metropolitan areas and their alignment with life expectancy and COVID-19 adjusted life expectancy. Our findings show a markedly unequal distribution of healthcare resources during the pandemic, whereby metropolitan areas with greater availability of resources tended to exhibit more favorable health outcomes. According to the World Health Organization (WHO) criteria (23), CI values equal to or greater than 0.2 indicate high levels of inequity. Under this criterion, our results reveal substantial inequities, particularly for resources consistently aligned with better health outcomes, such as specialized services, technological equipment, and health personnel in training. These are precisely the types of resources that should be more evenly distributed across metropolitan areas to advance toward a more equitable healthcare system. However, these findings should be interpreted as descriptive evidence of how the distribution of health resources corresponds with life expectancy outcomes across metropolitan areas, rather than as evidence of causal or independent associations between health resources and health outcomes.

Reductions in years of life in the 60–64 age group have also been observed in other countries (24). This decline is not surprising, given that COVID-19 mortality increased sharply with age and had a particularly severe consequences for populations living with chronic diseases (16). However, the impact on mortality in this age group in Mexico has been substantial. Before the outbreak, older adults aged 60 to 64 had a LE above 20 years (25). However, by 2020, this indicator declined substantially. Moreover, the high burden of chronic conditions among older adults in Mexico (26), likely contributed to amplifying the mortality impact of COVID-19. Additionally, our results show that this loss was rapidly reversed, with LE reaching 18.10 years in 2023, although it remained below pre-pandemic levels. A similar rapid recovery in LE was observed following previous health crises, such as the Spanish flu epidemic of 1918 to 1920 (2).

Concerning territorial analyses, previous evidence for Mexico showed that the impact of the pandemic on LE at birth exhibited high heterogeneity across states (4). Although this represents a higher level of aggregation than in our study, we likewise observed substantial variation, with a faster recovery of LE in the northern and central regions. This pattern is possibly linked to the long-standing economic development of these areas since the 1940s, which contrasts with the persistent structural disadvantages of southern states, including higher poverty levels, limited access to basic public services, higher illiteracy rates, and larger marginalized populations (27). These contextual conditions may have constrained their capacity to recover from the pandemic’s mortality impact.

The small differences between LE and CALE may reflect the high lethality of COVID-19 in this age group, whereby many individuals died before experiencing prolonged disability. Moreover, research assessing COVID-19 disability using disability-adjusted life years (DALYs) has shown that its impact has been concentrated primarily among males and older adults in Brazil (28), US (29), and Mexico (30).

Regarding the relationship between health resources and LE and CALE, physicians in training were the only variable consistently related with these indicators across all years, highlighting the strong reliance of the Mexican health system on trainees during the pandemic. In addition, resources related to specialized medical care showed significant relationships, in contrast to those linked to primary care. This pattern is expected, as COVID-19 cases requiring public health services were predominantly referred to specialized care due to their severity. This interpretation is supported by the relation observed between specialist physicians and DALE, suggesting that specialist availability may contribute to improved survival. The most consistent negative correlation was with emergency departments. This pattern likely reflects case severity rather than a detrimental effect of these services. Emergency departments tend to receive patients with more critical presentations and higher mortality risk, concentrating fatal and complex cases. Moreover, 2021 was the year in which the most consistent relations were observed, indicating that health system resources exerted their greatest influence on COVID-19 related mortality and disability during this year. As shown in our results, these relations weakened over time as the pandemic subsided.

Previous evidence has consistently shown that health resources are related with lower mortality and higher LE. In China, rises in the number of health technicians per 1,000 inhabitants had an increase on LE (17). While in Japan, a positive correlation was observed between the availability of therapists, support hospitals for home health care, and health system expenditure with LE and HALE (15). Similarly, in Indonesia, hospital beds as well as general and specialist physicians were related with higher LE (31).

Moreover, the unequal distribution of health resources across regions may contribute to unequal health outcomes. For instance, a study conducted in Japan found significant inequities in the distribution of health resources among the 344 secondary medical areas, with better-resourced areas concentrated in the central region of the country, in contrast to the northern regions (15). Similarly, in China, the most economically developed provinces consistently received the greatest allocation of health resources over time, generating marked disparities between northern and southern regions (17).

Research on LE and CALE in the context of COVID-19 considering small territorial units of analysis remains limited. However, a study conducted in Brazil using 558 microregions found that the availability of physicians and intensive care units in each municipality significantly affected COVID-19 mortality. The same study reported that northern states concentrated a higher risk of infection and mortality among older adults compared with southern states, where economic conditions are stronger due to tourism and business activity and where health resources are more available (16). In the case of Mexico, evidence from Mexico City shows that neighborhoods with low or very low values of the Social Development Index (SDI) experienced higher COVID-19 mortality rates compared with those with higher SDI levels (32). Additionally, studies have shown that municipalities with higher levels of marginalization are more likely to experience adverse COVID-19 outcomes, such as hospitalization and death (33).

On the other hand, during the pandemic, health systems were required to adapt rapidly to the uncertainty of the public health emergency, and this adaptive capacity became a key determinant of effective patient care. This process reflects health system resilience, understood as the ability of health institutions to respond effectively to abrupt changes in the population’s epidemiological profile while maintaining essential routine services. Resource availability and reallocation became central components of health system resilience. During the COVID-19 pandemic, across countries, new medical facilities were built, public spaces were converted into temporary hospitals, the health workforce was expanded through the incorporation of medical and nursing students or the re-engagement of retired physicians, and supply chains were strengthened (14). These strategies involved the redistribution of existing resources.

In Mexico, similar measures were implemented; however, these were largely reactive rather than preventive. As the COVID-19 pandemic health impacts became evident, government responses were primarily oriented toward mitigating its consequences rather than interrupting transmission. This resulted in unplanned and heterogeneous strategies to address its effects (34). For example, interventions focused on increasing hospital capacity (35). In addition, the health system was undergoing a major structural reform at the onset of the pandemic, including the elimination of *Seguro Popular* (a program that provided health resources for the uninsured population) and the creation of the Health Institute for Welfare (*Instituto de Salud para el Bienestar*, INSABI). However, INSABI faced significant challenges in delivering services to this population and was ultimately dissolved in 2023 (36). This reform generated considerable uncertainty, as it lacked clear operational rules and resource allocation mechanisms, further weakening the system’s capacity to respond effectively. These institutional changes occurred alongside pre-existing structural limitations of the Mexican health system, including service saturation, low public health expenditure, and insufficient health personnel and infrastructure. For instance, the number of physicians, nurses, and hospital beds remains below Organisation for Economic Co-operation and Development (OECD) members averages. As a result, Mexico experienced a particularly high mortality impact during the pandemic (35).

Moreover, these reactive strategies focused on increasing hospital capacity and reconverting health facilities to treat COVID-19 patients (35) may not have occurred equitably across territories. Accordingly, using the DI, we identified that several resources require redistribution, particularly high-technology equipment, emergency services, specialist physicians, medical offices, and physicians in training. Previous research has also documented this imbalance in the distribution of health professionals, indicating that 12.6% of the health workforce would need to be redistributed to achieve the national average of health personnel across states, given that Mexico City, Nuevo León, Puebla, and Jalisco concentrate more than 40% of health personnel (37).

Resource allocation may be guided by two main ethical perspectives: egalitarianism and utilitarianism. The egalitarian approach proposes that benefits should be available to everyone, based on the principle that all individuals are equal and should not be subject to segregation. In contrast, utilitarianism argues that public health outcomes should be maximized by prioritizing overall social welfare (38). Our findings do not allow us to determine which perspective should be followed, given the complexity of this decision. Nevertheless, they clearly indicate that redistribution is necessary and that further research is required to determine how and where resources should be reallocated. This information is essential for improving preparedness for future infectious disease emergencies. May be, a balanced approach is required, combining both perspectives by prioritizing the most vulnerable and disadvantaged populations or territories, while avoiding discriminatory criteria, such as age, socioeconomic status, or ethnicity, that could restrict access to medical care (39).

We present an unexplored assessment of inequities in the distribution of health resources across metropolitan areas in relation to LE, CALE, and DALE during the COVID-19 pandemic. Nevertheless, several limitations should be acknowledged. First, the analysis focused exclusively on older adults from 60 to 64 age group; however, other age groups should be examined in future research. Second, under registration of COVID-19 cases and deaths among older adults is possible, as many individuals may not have accessed public health services, sought care, remained at home or in rural areas, or may have been misclassified under other causes of death. Third, although HALE was used as an indirect measure of quality of life, the impact of COVID-19 is long term, particularly regarding respiratory function and mental health. Due to data limitations, long COVID could not be assessed, and only immediate morbidity was evaluated. Fourth, health resource data were obtained exclusively from public health services; information from private providers was unavailable and therefore could not be included. Fifth, the types of health resources analyzed were limited to those available in the database and did not include certain resources that were critical during the COVID-19 emergency, such as oxygen equipment and personal protective equipment. Finally, socioeconomic variables would be desirable to include, as the health system represents only part of the broader context. However, information on income, governmental support, and related factors was not available for the period and the territorial level of analysis. Consequently, the results should be interpreted with caution, as they were estimated without adjustment for potential socioeconomic confounders.

## Conclusion

The impact of the COVID-19 pandemic on older adults’ health was substantial. Health system adaptations were crucial to addressing this public health emergency, particularly regarding the territorial distribution of resources. Our findings indicate that this redistribution process was not equitable across Mexican metropolitan areas, as those with greater resource availability tended to exhibit higher life expectancy and COVID-19 adjusted life expectancy. Resources aligned with more favorable health outcomes were primarily related to specialized services, technological equipment, and health personnel in training. Redistributing these resources is necessary to move toward a more equitable health system and to better confront future public health crises. However, these findings should be interpreted as descriptive evidence of how the distribution of health resources corresponds with health outcomes across metropolitan areas, rather than as evidence of causal or independent relationships.

## Data Availability

Publicly available datasets were analyzed in this study. This data can be found at: Mortality data from the National Institute of Statistics and Geography (https://www.inegi.org.mx/programas/edr/#microdatos), mid-year population estimates from the National Population Council (https://www.datos.gob.mx/dataset/proyecciones-de-poblacion), COVID-19 case data (https://www.gob.mx/salud/documentos/datos-abiertos-bases-historicas-direccion-general-de-epidemiologia) and information on health resources (http://www.dgis.salud.gob.mx/contenidos/basesdedatos/da_recursos_gobmx.html) from General Directorate of Epidemiology.
