# Supplementary material for: Metropolitan inequalities in health system resources and COVID-19 adjusted life expectancy among older adults in Mexico
Source: Front Public Health. 2026 May 26;14:1807629. doi: 10.3389/fpubh.2026.1807629 (PMC13246358; doi:10.3389/fpubh.2026.1807629)
Supplement: Supplementary file 1 [file Table_1.DOCX]

Supplementary material 1. Life expectancy (LE), COVID-19 adjusted life expectancy (CALE), and COVID-19 disability-adjusted life expectancy (DALE) at ages 60-64 across Mexican metropolitan areas, 2020 to 2023.

|  | 2020 | | | 2021 | | | 2022 | | | 2023 | | |
| --- | --- | --- | --- | --- | --- | --- | --- | --- | --- | --- | --- | --- |
| Metropolitan area | LE | CALE | DALE | LE | CALE | DALE | LE | CALE | DALE | LE | CALE | DALE |
| Aguascalientes | 14.09 | 13.80 | 0.29 | 14.26 | 13.94 | 0.32 | 16.74 | 16.29 | 0.45 | 18.34 | 18.25 | 0.10 |
| Tijuana | 13.07 | 12.84 | 0.23 | 14.69 | 14.47 | 0.22 | 16.72 | 16.53 | 0.19 | 18.38 | 18.33 | 0.05 |
| Ensenada | 12.66 | 12.31 | 0.35 | 12.86 | 12.49 | 0.37 | 15.80 | 15.44 | 0.36 | 17.74 | 17.64 | 0.10 |
| Mexicali | 11.02 | 10.70 | 0.32 | 14.16 | 13.84 | 0.32 | 17.11 | 16.85 | 0.26 | 17.87 | 17.81 | 0.05 |
| La Paz | 14.87 | 14.39 | 0.48 | 14.16 | 13.36 | 0.80 | 16.38 | 15.02 | 1.36 | 18.16 | 17.98 | 0.18 |
| Los Cabos | 13.28 | 12.95 | 0.33 | 13.27 | 12.73 | 0.54 | 16.60 | 15.85 | 0.75 | 17.60 | 17.50 | 0.11 |
| Campeche | 13.98 | 13.74 | 0.25 | 13.98 | 13.55 | 0.42 | 18.49 | 18.03 | 0.45 | 17.88 | 17.85 | 0.04 |
| La Laguna | 11.45 | 11.11 | 0.33 | 15.05 | 14.82 | 0.23 | 16.06 | 15.83 | 0.22 | 17.52 | 17.47 | 0.05 |
| Monclova-Frontera | 11.76 | 11.36 | 0.40 | 14.00 | 13.72 | 0.28 | 15.39 | 15.04 | 0.35 | 16.96 | 16.86 | 0.10 |
| Piedras Negras | 11.61 | 11.19 | 0.42 | 11.81 | 11.52 | 0.29 | 13.94 | 13.61 | 0.33 | 15.09 | 15.02 | 0.07 |
| Saltillo | 12.25 | 11.99 | 0.26 | 13.76 | 13.50 | 0.26 | 15.91 | 15.59 | 0.32 | 17.48 | 17.40 | 0.07 |
| Sabinas | 11.72 | 11.24 | 0.48 | 12.44 | 12.04 | 0.40 | 14.38 | 14.08 | 0.30 | 15.40 | 15.29 | 0.12 |
| Colima-Villa de Álvarez | 16.08 | 15.82 | 0.27 | 14.42 | 13.95 | 0.47 | 17.34 | 16.68 | 0.66 | 17.89 | 17.73 | 0.16 |
| Tecomán | 13.38 | 13.16 | 0.23 | 11.78 | 11.42 | 0.36 | 16.08 | 15.84 | 0.24 | 16.65 | 16.64 | 0.01 |
| Tapachula | 11.89 | 11.80 | 0.10 | 11.89 | 11.77 | 0.12 | 15.12 | 14.98 | 0.14 | 15.98 | 15.96 | 0.02 |
| Tuxtla Gutiérrez | 11.93 | 11.81 | 0.12 | 13.51 | 13.41 | 0.10 | 16.49 | 16.34 | 0.15 | 17.03 | 17.01 | 0.02 |
| Chihuahua | 12.89 | 12.66 | 0.24 | 14.11 | 13.81 | 0.30 | 15.81 | 15.44 | 0.37 | 17.09 | 17.05 | 0.04 |
| Delicias | 11.73 | 11.52 | 0.22 | 13.13 | 12.93 | 0.20 | 14.83 | 14.65 | 0.18 | 17.15 | 17.12 | 0.03 |
| Juárez | 9.40 | 9.17 | 0.24 | 14.32 | 14.15 | 0.16 | 14.65 | 14.43 | 0.22 | 16.04 | 16.01 | 0.03 |
| Hidalgo del Parral | 12.50 | 12.22 | 0.28 | 14.76 | 14.40 | 0.35 | 16.04 | 15.41 | 0.63 | 18.38 | 18.34 | 0.04 |
| Ciudad de México | 12.72 | 12.30 | 0.42 | 13.42 | 12.89 | 0.54 | 17.82 | 17.10 | 0.72 | 18.84 | 18.66 | 0.18 |
| Durango | 14.25 | 13.87 | 0.38 | 14.54 | 14.30 | 0.25 | 16.48 | 16.20 | 0.28 | 18.47 | 18.41 | 0.07 |
| Celaya | 13.27 | 12.94 | 0.33 | 12.87 | 12.59 | 0.28 | 16.23 | 15.96 | 0.27 | 16.86 | 16.79 | 0.07 |
| León | 13.05 | 12.66 | 0.39 | 12.56 | 12.18 | 0.38 | 16.12 | 15.60 | 0.52 | 17.27 | 17.18 | 0.09 |
| Guanajuato | 14.14 | 13.78 | 0.37 | 12.95 | 12.51 | 0.44 | 16.47 | 16.00 | 0.47 | 17.15 | 17.07 | 0.08 |
| Irapuato | 13.15 | 12.81 | 0.35 | 12.42 | 12.09 | 0.33 | 16.06 | 15.69 | 0.37 | 16.87 | 16.81 | 0.06 |
| Moroleón-Uriangato | 15.47 | 15.14 | 0.33 | 14.15 | 13.90 | 0.24 | 17.94 | 17.69 | 0.25 | 18.38 | 18.32 | 0.06 |
| Silao | 12.69 | 12.36 | 0.33 | 12.86 | 12.60 | 0.26 | 16.56 | 16.38 | 0.18 | 17.75 | 17.72 | 0.03 |
| Chilpancingo | 14.88 | 14.46 | 0.42 | 15.07 | 14.66 | 0.41 | 19.13 | 18.65 | 0.48 | 21.17 | 21.08 | 0.09 |
| Acapulco | 13.70 | 13.37 | 0.33 | 13.16 | 12.75 | 0.40 | 18.54 | 18.18 | 0.36 | 18.77 | 18.71 | 0.06 |
| Pachuca | 15.17 | 14.78 | 0.39 | 14.69 | 14.39 | 0.30 | 18.71 | 18.10 | 0.61 | 19.28 | 19.10 | 0.17 |
| Tulancingo | 13.77 | 13.51 | 0.26 | 12.50 | 12.18 | 0.33 | 17.08 | 16.69 | 0.39 | 18.33 | 18.16 | 0.16 |
| Atitalaquia | 13.21 | 12.86 | 0.36 | 13.21 | 12.98 | 0.23 | 17.47 | 17.30 | 0.17 | 18.72 | 18.69 | 0.03 |
| Guadalajara | 14.66 | 14.42 | 0.24 | 13.42 | 13.18 | 0.24 | 16.82 | 16.55 | 0.27 | 18.05 | 18.00 | 0.05 |
| Puerto Vallarta | 13.18 | 12.95 | 0.23 | 12.65 | 12.28 | 0.37 | 16.28 | 15.99 | 0.28 | 16.84 | 16.77 | 0.06 |
| Ocotlán | 14.94 | 14.77 | 0.17 | 12.18 | 11.93 | 0.25 | 16.10 | 15.96 | 0.14 | 16.95 | 16.91 | 0.04 |
| Toluca | 11.57 | 11.33 | 0.23 | 12.75 | 12.60 | 0.14 | 17.07 | 16.91 | 0.16 | 18.07 | 18.01 | 0.06 |
| Ozumba | 12.04 | 11.91 | 0.13 | 10.67 | 10.55 | 0.12 | 16.16 | 16.01 | 0.15 | 17.93 | 17.90 | 0.03 |
| Tianguistenco | 9.04 | 8.80 | 0.25 | 10.31 | 10.13 | 0.18 | 14.46 | 14.30 | 0.16 | 16.26 | 16.21 | 0.05 |
| La Piedad-Pénjamo | 14.20 | 13.95 | 0.24 | 12.74 | 12.56 | 0.18 | 16.68 | 16.57 | 0.11 | 17.70 | 17.68 | 0.02 |
| Morelia | 17.60 | 17.32 | 0.28 | 14.76 | 14.54 | 0.21 | 19.02 | 18.78 | 0.24 | 19.70 | 19.57 | 0.13 |
| Zamora | 14.79 | 14.65 | 0.15 | 12.85 | 12.69 | 0.15 | 17.24 | 17.11 | 0.13 | 17.75 | 17.71 | 0.04 |
| Uruapan | 15.47 | 15.25 | 0.21 | 14.90 | 14.68 | 0.22 | 18.69 | 18.53 | 0.16 | 19.53 | 19.48 | 0.06 |
| Lázaro Cárdenas | 13.81 | 13.33 | 0.48 | 14.00 | 13.48 | 0.52 | 18.68 | 18.42 | 0.27 | 19.00 | 18.98 | 0.03 |
| Sahuayo | 14.78 | 14.62 | 0.16 | 13.70 | 13.56 | 0.14 | 17.05 | 16.91 | 0.14 | 16.53 | 16.51 | 0.02 |
| Cuautla | 13.42 | 13.23 | 0.19 | 12.06 | 11.72 | 0.34 | 16.23 | 15.92 | 0.31 | 17.28 | 17.20 | 0.08 |
| Cuernavaca | 14.76 | 14.59 | 0.16 | 13.04 | 12.70 | 0.34 | 16.75 | 16.48 | 0.27 | 17.68 | 17.61 | 0.07 |
| Tepic | 16.04 | 15.76 | 0.28 | 14.58 | 14.15 | 0.43 | 18.03 | 17.34 | 0.69 | 19.46 | 19.26 | 0.20 |
| Monterrey | 14.09 | 13.74 | 0.34 | 14.27 | 13.95 | 0.31 | 16.45 | 16.04 | 0.41 | 17.52 | 17.40 | 0.11 |
| Oaxaca | 15.08 | 14.49 | 0.58 | 14.25 | 13.69 | 0.56 | 17.81 | 16.92 | 0.89 | 18.77 | 18.54 | 0.22 |
| Juchitán de Zaragoza | 13.89 | 13.78 | 0.11 | 12.01 | 11.79 | 0.21 | 17.52 | 17.33 | 0.19 | 18.97 | 18.93 | 0.05 |
| Salina Cruz | 13.17 | 12.95 | 0.22 | 11.18 | 10.84 | 0.35 | 15.26 | 14.87 | 0.39 | 15.66 | 15.62 | 0.05 |
| Tehuantepec | 12.33 | 12.21 | 0.11 | 10.73 | 10.57 | 0.16 | 14.71 | 14.57 | 0.14 | 15.40 | 15.38 | 0.02 |
| Puebla-Tlaxcala | 12.57 | 12.24 | 0.33 | 11.89 | 11.55 | 0.33 | 16.78 | 16.46 | 0.33 | 17.66 | 17.55 | 0.11 |
| San Martín Texmelucan | 11.96 | 11.73 | 0.24 | 11.13 | 10.98 | 0.15 | 16.04 | 15.91 | 0.13 | 16.90 | 16.87 | 0.03 |
| Tehuacán | 12.68 | 12.49 | 0.19 | 11.42 | 11.22 | 0.20 | 16.48 | 16.32 | 0.16 | 17.11 | 17.07 | 0.04 |
| Huauchinango | 13.01 | 12.84 | 0.17 | 12.18 | 12.01 | 0.18 | 15.89 | 15.67 | 0.22 | 15.99 | 15.95 | 0.04 |
| Teziutlán | 12.99 | 12.75 | 0.24 | 12.27 | 12.01 | 0.26 | 15.55 | 15.31 | 0.24 | 16.01 | 15.96 | 0.05 |
| Querétaro | 15.12 | 14.62 | 0.50 | 14.21 | 13.74 | 0.47 | 17.30 | 16.77 | 0.53 | 18.72 | 18.58 | 0.14 |
| Cancún | 12.42 | 12.15 | 0.27 | 13.11 | 12.78 | 0.33 | 16.48 | 16.04 | 0.44 | 16.96 | 16.91 | 0.05 |
| Chetumal | 13.24 | 12.89 | 0.36 | 13.65 | 13.29 | 0.36 | 16.77 | 16.47 | 0.30 | 17.04 | 17.00 | 0.05 |
| Playa del Carmen | 13.44 | 13.14 | 0.31 | 14.18 | 13.82 | 0.36 | 17.45 | 17.12 | 0.33 | 16.59 | 16.55 | 0.04 |
| San Luis Potosí | 13.10 | 12.62 | 0.48 | 13.98 | 13.47 | 0.51 | 17.07 | 15.88 | 1.19 | 18.00 | 17.82 | 0.18 |
| Matehuala | 14.63 | 14.23 | 0.40 | 15.42 | 14.89 | 0.54 | 16.40 | 15.40 | 1.00 | 19.30 | 19.22 | 0.08 |
| Rioverde | 15.46 | 15.22 | 0.24 | 14.54 | 14.07 | 0.47 | 16.94 | 16.42 | 0.52 | 18.06 | 18.02 | 0.04 |
| Culiacán | 14.96 | 14.62 | 0.34 | 15.66 | 15.33 | 0.33 | 18.94 | 17.93 | 1.01 | 20.15 | 20.02 | 0.13 |
| Los Mochis | 14.08 | 13.84 | 0.24 | 16.18 | 15.90 | 0.27 | 19.81 | 19.23 | 0.58 | 20.94 | 20.87 | 0.06 |
| Mazatlán | 13.67 | 13.43 | 0.24 | 14.13 | 13.90 | 0.24 | 17.60 | 17.08 | 0.52 | 18.10 | 18.05 | 0.06 |
| Guaymas | 12.03 | 11.68 | 0.35 | 12.44 | 12.08 | 0.36 | 15.36 | 15.12 | 0.24 | 16.31 | 16.29 | 0.03 |
| Ciudad Obregón | 12.98 | 12.61 | 0.37 | 14.56 | 14.08 | 0.48 | 17.00 | 16.60 | 0.40 | 18.03 | 17.97 | 0.06 |
| Hermosillo | 12.75 | 12.13 | 0.62 | 14.73 | 14.27 | 0.45 | 16.76 | 16.33 | 0.43 | 18.31 | 18.26 | 0.05 |
| Nogales | 11.44 | 11.02 | 0.42 | 13.78 | 13.54 | 0.23 | 15.51 | 15.17 | 0.34 | 16.47 | 16.41 | 0.05 |
| Caborca | 11.95 | 11.45 | 0.50 | 14.49 | 14.02 | 0.47 | 15.32 | 14.92 | 0.40 | 17.77 | 17.70 | 0.07 |
| Villahermosa | 11.52 | 10.95 | 0.57 | 14.17 | 13.32 | 0.85 | 16.89 | 15.89 | 1.00 | 17.10 | 17.06 | 0.05 |
| Reynosa | 11.60 | 11.40 | 0.20 | 14.32 | 14.12 | 0.21 | 15.53 | 15.28 | 0.25 | 16.53 | 16.49 | 0.03 |
| Tampico | 15.19 | 14.85 | 0.34 | 16.51 | 16.11 | 0.40 | 18.73 | 18.18 | 0.54 | 20.13 | 20.08 | 0.06 |
| Ciudad Victoria | 16.55 | 16.17 | 0.37 | 16.16 | 15.83 | 0.33 | 18.96 | 18.65 | 0.30 | 19.28 | 19.22 | 0.06 |
| Matamoros | 14.47 | 14.19 | 0.28 | 15.78 | 15.55 | 0.23 | 17.88 | 17.58 | 0.30 | 18.13 | 18.09 | 0.04 |
| Nuevo Laredo | 13.23 | 13.01 | 0.22 | 13.85 | 13.65 | 0.20 | 15.70 | 15.55 | 0.15 | 17.21 | 17.17 | 0.04 |
| Tlaxcala | 12.98 | 12.68 | 0.29 | 12.74 | 12.47 | 0.27 | 17.93 | 17.62 | 0.31 | 18.56 | 18.45 | 0.11 |
| Huamantla | 13.18 | 13.04 | 0.15 | 12.93 | 12.75 | 0.19 | 17.36 | 17.13 | 0.23 | 17.51 | 17.45 | 0.06 |
| Coatzacoalcos | 12.04 | 11.85 | 0.19 | 12.59 | 12.33 | 0.26 | 16.19 | 16.00 | 0.19 | 16.71 | 16.68 | 0.03 |
| Córdoba | 13.57 | 13.40 | 0.17 | 14.28 | 14.08 | 0.20 | 16.04 | 15.78 | 0.26 | 16.75 | 16.65 | 0.09 |
| Minatitlán | 13.16 | 13.01 | 0.15 | 12.54 | 12.36 | 0.18 | 15.94 | 15.83 | 0.11 | 16.56 | 16.55 | 0.01 |
| Orizaba | 13.56 | 13.34 | 0.22 | 13.67 | 13.45 | 0.22 | 16.19 | 15.92 | 0.27 | 17.01 | 16.94 | 0.08 |
| Poza Rica | 13.42 | 13.17 | 0.25 | 13.27 | 13.05 | 0.22 | 17.02 | 16.80 | 0.22 | 17.02 | 16.98 | 0.03 |
| Veracruz | 13.32 | 13.02 | 0.29 | 14.73 | 14.47 | 0.27 | 17.17 | 16.84 | 0.33 | 17.30 | 17.23 | 0.07 |
| Xalapa | 15.43 | 15.28 | 0.15 | 15.36 | 15.15 | 0.21 | 17.51 | 17.12 | 0.39 | 17.69 | 17.57 | 0.12 |
| Acayucan | 13.36 | 13.30 | 0.06 | 11.31 | 11.23 | 0.08 | 16.12 | 16.10 | 0.02 | 16.94 | 16.94 | 0.00 |
| Mérida | 14.56 | 14.26 | 0.29 | 14.93 | 14.53 | 0.40 | 17.58 | 17.12 | 0.46 | 18.06 | 18.00 | 0.06 |
| Valladolid | 12.97 | 12.55 | 0.43 | 12.62 | 12.31 | 0.31 | 16.59 | 16.35 | 0.24 | 17.31 | 17.29 | 0.03 |
| Zacatecas-Guadalupe | 13.43 | 12.87 | 0.56 | 14.65 | 14.21 | 0.44 | 16.22 | 15.49 | 0.73 | 17.54 | 17.38 | 0.15 |
